# Thyroid Hormone Signaling in the Mouse Retina

**DOI:** 10.1371/journal.pone.0168003

**Published:** 2016-12-12

**Authors:** Patrick Arbogast, Frédéric Flamant, Pierre Godement, Martin Glösmann, Leo Peichl

**Affiliations:** 1 Max Planck Institute for Brain Research, Max-von-Laue-Str., Frankfurt, Germany; 2 Institut de Génomique Fonctionnelle de Lyon, Ecole Normale Supérieure de Lyon, 46 Allée d’Italie, Lyon CEDEX 07, France; 3 VetCore/Imaging, University of Veterinary Medicine, Veterinärplatz 1, Vienna, Austria; 4 Ernst Strüngmann Institute for Neuroscience, Deutschordenstr.,Frankfurt, Germany; National Eye Centre, UNITED STATES

## Abstract

Thyroid hormone is a crucial regulator of gene expression in the developing and adult retina. Here we sought to map sites of thyroid hormone signaling at the cellular level using the transgenic FINDT3 reporter mouse model in which neurons express β-galactosidase (β-gal) under the control of a hybrid Gal4-TRα receptor when triiodothyronine (T3) and cofactors of thyroid receptor signaling are present. In the adult retina, nearly all neurons of the ganglion cell layer (GCL, ganglion cells and displaced amacrine cells) showed strong β-gal labeling. In the inner nuclear layer (INL), a minority of glycineric and GABAergic amacrine cells showed β-gal labeling, whereas the majority of amacrine cells were unlabeled. At the level of amacrine types, β-gal labeling was found in a large proportion of the glycinergic AII amacrines, but only in a small proportion of the cholinergic/GABAergic ‘starburst’ amacrines. At postnatal day 10, there also was a high density of strongly β-gal-labeled neurons in the GCL, but only few amacrine cells were labeled in the INL. There was no labeling of bipolar cells, horizontal cells and Müller glia cells at both stages. Most surprisingly, the photoreceptor somata in the outer nuclear layer also showed no β-gal label, although thyroid hormone is known to control cone opsin expression. This is the first record of thyroid hormone signaling in the inner retina of an adult mammal. We hypothesize that T3 levels in photoreceptors are below the detection threshold of the reporter system. The topographical distribution of β-gal-positive cells in the GCL follows the overall neuron distribution in that layer, with more T3-signaling cells in the ventral than the dorsal half-retina.

## Introduction

Thyroid hormone (TH), particularly in its biologically active form triiodothyronine (T3), plays an important role in brain development and various brain functions; this also includes the retina (see, e.g., [1–3]). The thyroid gland secrets the precursor hormone thyroxine (T4) and some T3, which is provided to tissues by the blood serum. In the organs, T3 levels are tissue- and cell-specifically regulated by the deiodinases (reviews: [4–6]). T3 acts via the TH receptors TRα and TRβ, which are ligand-dependent nuclear transcription factors that regulate gene expression (reviews: [7–8]).

In the retina, a number of developmental mechanisms depend on the presence of TRs and TH, and both TR isoforms, alpha and beta, are known to be expressed in the vertebrate retina (e.g., [2,7,9–10]). Prenatally, TH is an important regulator in the normal development of the eye and retina [11–14]. Postnatally, TH is crucial for the differentiation of spectral cone types. The majority of mammalian species possess two types of retinal cone photoreceptors, characterized by the expression of either a shortwave-sensitive (S) cone opsin or a middle- to longwave-sensitive (M) cone opsin (reviews: [15–16]). By default, cones express S opsin [17–18]. For M cone differentiation, TH signaling is required via TRβ2, a receptor that in the retina is expressed exclusively in the cones [19–21]. Knockout mice missing TRβ2 develop no M cones, and all cones express the S opsin [19]. The presence of TH is required for this TRβ2 action, as transgenic mice with a ligand binding-defective TRβ2 as well as hypothyroid mice show a similar cone opsin expression pattern as TRβ2^-/-^ mice [21–23]. In mice M opsin expression starts in the second postnatal week around p10 concomitant with elevated TH levels in the dorsal retina [24] and TH remains relevant for opsin regulation even after terminal maturation of the cones, as pharmacological suppression of TH in adult wildtype mice results in decreased M opsin levels and increased S opsin levels [25]. TH also is involved in apoptotic processes in cones [26–27].

To map sites of thyroid hormone signaling in the adult and developing mouse retina, we used transgenic FINDT3 reporter mice [28] in which neurons express β-galactosidase in the presence of T3. The reporter plasmid encodes for its own thyroid hormone receptor and is therefore independent of endogenous TRs. Otherwise TH signaling is unaltered compared to wildtype mouse. This approach allowed us to localize T3 signaling at the level of individual cells, and it yielded some unexpected results.

## Materials and Methods

### Ethics Statement

All procedures for animal husbandry, breeding and killing complied with the NIH Principles of Laboratory Animal Care and the European Communities Council Directives of November 24, 1986 (86/609/EEC) and September 22, 2010 (2010/63/EU) regarding the protection of animals used for experimental and other scientific purposes. The initial research project had been approved by a local animal care and use committee at the Lyon institute and subsequently authorized by the French Ministry of Research. Mice were bred and maintained at the Plateau de Biologie Expérimentale de la Souris (SFR BioSciences Gerland—Lyon Sud, France). Animals were killed by decapitation under deep isoflurane anesthesia.

### Animals and tissue preparation

Transgenic FINDT3 reporter mice (line FINDT3B) were generated at the Institut de Génomique Fonctionnelle de Lyon, France [28]. Two adult (one month old) and two ten day old (p10) FINDT3 animals and two adult wildtype control animals from the same colony were used for the study. Immediately post mortem, eyes were marked at the dorsal pole for orientation, enucleated, punctured at the corneal rim for better fixative penetration, and immersion-fixed in 4% paraformaldehyde in 0.1 M phosphate buffer (PB, pH 7.4) for between 30 min and 1 h at room temperature. After a wash in PB, the retina was isolated from the eyecup and either processed immediately, or cryo-protected by successive immersion in 10%, 20% and 30% (w/v) sucrose in PB and frozen at -20°C for later use.

For frozen vertical sections of the retina (i.e., perpendicular to the retinal layers) the tissue was cryoprotected by successive immersion in 10%, 20% and 30% (w/v) sucrose in PB, transferred to tissue-freezing medium (Reichert-Jung, Bensheim, Germany), frozen, sectioned at a thickness of 12–14 μm with a cryostat, and collected on Superfrost Plus slides (Menzel Gläser, Braunschweig, Germany).

### Immunostaining and histochemistry

Immunostaining was performed on frozen sections following previously described protocols [29–30]. Briefly, the tissue was preincubated for 1 h in PBS with 0.5% Triton X-100 and 10% normal donkey serum (NDS). Incubation in the primary antibody/antiserum solution was overnight at room temperature. β-galactosidase (β-gal) was detected with the mouse monoclonal antibody G8021 (dilution 1:500; Sigma-Aldrich, St. Louis, USA) or a rabbit polyclonal antiserum (dilution 1:500; Cat. No. 5307–063100, 5 Prime → 3 Prime, West Chester, USA). Double labeling of sections with these two antibodies showed complete colocalization of the two labels, documenting that both had the same specificity for β-gal. In double immunofluorescence labelings for β-gal and amacrine cells, we used the rabbit polyclonal antiserum A2051 against GABA (dilution 1:2000; Sigma-Aldrich, St. Louis, USA) to label GABAergic amacrines, the goat polyclonal antiserum AB1770 against glycin transporter 1 (GlyT1, dilution 1:10.000; Chemicon, Temecula, USA) to label glycinergic amacrines, a rabbit polyclonal antiserum against Disabled-1 (Dab1, dilution 1:1000; kindly provided by B. Howell, NIH [31]) to label AII amacrines, and the goat polyclonal antiserum AB144P against choline acetyltransferase (ChAT; dilution 1:200; EMD Millipore, Darmstadt, Germany) to label cholinergic amacrines. Double labeling for β-gal and rod bipolar cells was performed using the rabbit polyclonal antiserum P4334 against protein kinase C alpha (PKCα; dilution 1:5000; Sigma-Aldrich, St. Louis, USA). Double labeling for β-gal and Müller glia cells was performed using the mouse monoclonal antibody 610518 against glutamine synthetase (dilution 1:500; BD Transduction Laboratories, Franklin Lakes, USA). Binding sites of the primary antibodies were detected by indirect immunofluorescence, by a 1 h incubation of the sections in secondary antisera from donkey conjugated to Alexa 488 or Cy5. For the double-labelings a mixture of 488-conjugated and Cy5-conjugated secondary antisera was used. Omission of the primary antibodies from the incubation solution resulted in no staining, except for the expected labeling of retinal blood vessels by secondary anti-mouse IgG antisera.

Histochemical X-gal staining was performed on whole freefloating retinae and on frozen sections, using the β-Galactosidase Reporter Gene Staining Kit (Sigma-Aldrich, St. Louis, USA). Tissue was incubated in staining solution (containing 1% magnesium chloride, 1% potassium ferricyanide, 1% potassium ferrocyanide and 5% X-gal) for 2 h– 1 day at 37°C. Whole retinae were flat-mounted on slides with the photoreceptor side facing down. All tissue was coverslipped with an aqueous mounting medium (AquaPoly/Mount, Polysciences Inc., Warrington, USA). Parallel staining of wildtype (negative) controls with the same incubation times showed no X-gal label at all, indicating that the staining is specific and without background.

### Imaging and analysis

Tissue was analyzed with a Zeiss Axiophot 2 microscope and a Zeiss Axioplan 2 equipped with epifluorescence. Micrographs were taken with a CCD camera and the Axiovision LE software (Carl Zeiss Vision, Germany). Some micrographs were taken with a laser scanning microscope Olympus FluoView 1000, using the FV 1.7 software (Olympus). The images were adjusted for brightness and contrast using Adobe Photoshop CS5.

To determine the density of X-gal-stained cells in the ganglion cell layer (GCL) and inner nuclear layer (INL) of flat-mounted retinae, counts were made in 250μm x 250μm sample fields with a 40x oil immersion objective, focused either on the GCL or the INL. Densities were not corrected for shrinkage, which was negligible in the tissues mounted with the aqueous medium.

## Results

**Fig 1:**
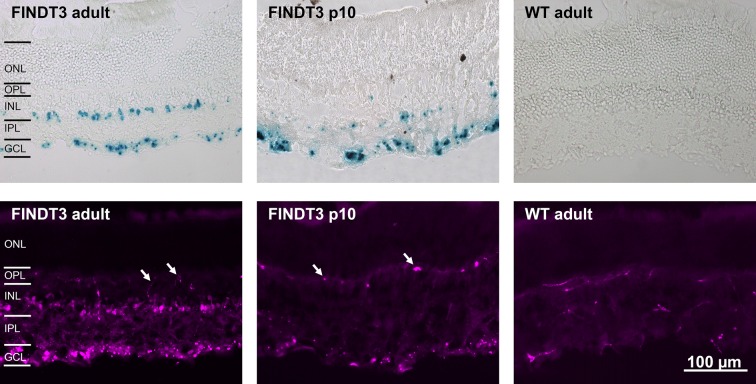
Vertical cryostat sections of FINDT3 and wildtype mouse retinae stained for β-galactosidase. **Top row:** X-gal staining, blue dots indicate β-gal-positive cells. In adult FINDT3 mice (left) staining occurs in the ganglion cell layer (GCL) and inner nuclear layer (INL), but not in the outer nuclear layer (ONL). In FINDT3 mice at postnatal day 10 (p10, middle) staining mainly occurs in the GCL with only few labeled cells in the INL. There is no staining in the adult wildtype control (WT, right). **Bottom row:** Staining with the mouse monoclonal antibody against β-gal. As in the X-gal staining, adult FINDT3 mice show two bands of stained cells in the GCL and INL, respectively. At p10 staining mainly occurs in the GCL with few labeled cells in the INL. In all these immunolabeled sections, blood vessels are also stained by the anti-mouse secondary antibody (some arrowed); in the wildtype control, the only labeled structures are blood vessels. OPL, outer plexiform layer; IPL, inner plexiform layer. Images were acquired with a Zeiss Axiophot 2 microscope (top row) and a Zeiss Axioplan 2 microscope (bottom row). The scale bar applies to all images.

The FINDT3 reporter mouse expresses β-gal in the presence of triiodothyronine (T3). It is based on an artificial Gal4-TRα fusion protein and does not depend on the expression of endogenous receptors. In vertical sections of adult retinae, X-gal staining showed two distinctive bands of stained cells, one in the ganglion cell layer (GCL) and one in the inner nuclear layer (INL; Fig 1). The photoreceptor somata in the outer nuclear layer (ONL) were not stained. At p10 the GCL was similarly stained, but the INL contained only few labeled cells (Fig 1). In both cases the INL staining only appeared after a few hours of incubation whereas the GCL staining was visible after less than one hour of incubation, indicating higher levels of β-gal in the GCL. No staining was observed in wildtype controls (Fig 1). Staining with antibodies against β-gal resulted in a similar staining pattern (Fig 1). In wildtype controls only blood vessels were stained. This label originated from the anti-mouse IgG secondary antibody, as evident in controls where the primary antibody was omitted.

**Fig 2:**
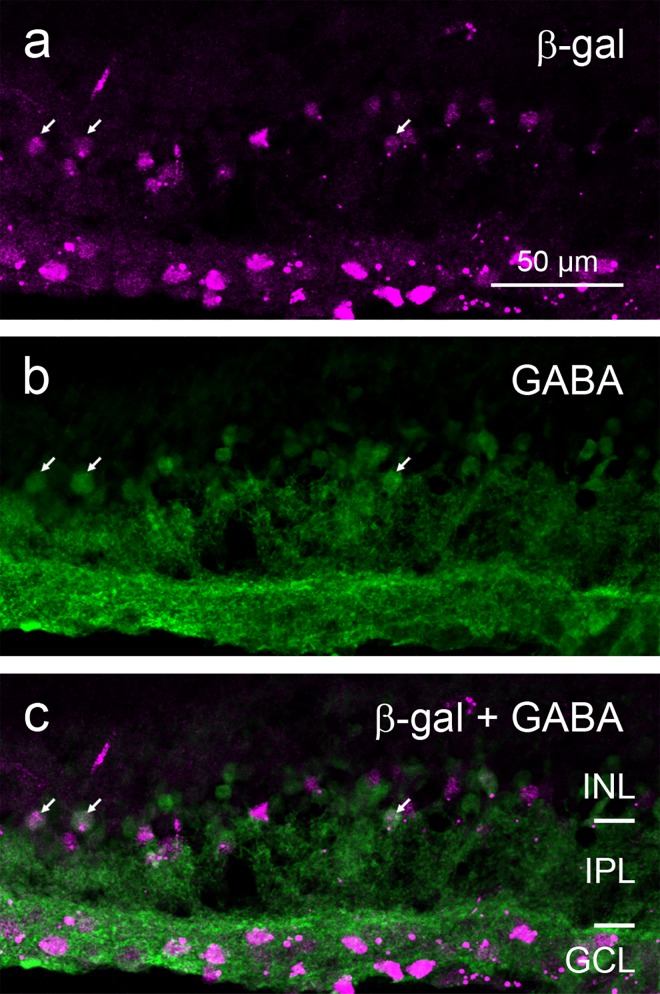
Double immunostaining of a vertical cryostat section of adult FINDT3 mouse retina for β-gal and GABAergic amacrine cells. β-gal label (**a**) colocalizes with the GABA label (**b**) in some somata in the INL (arrows point to examples), but many GABAergic amacrine cells show no β-gal signal, as evident in the merge (**c**). Images were acquired with an Olympus FluoView 1000 laser scanning microscope.

**Fig 3:**
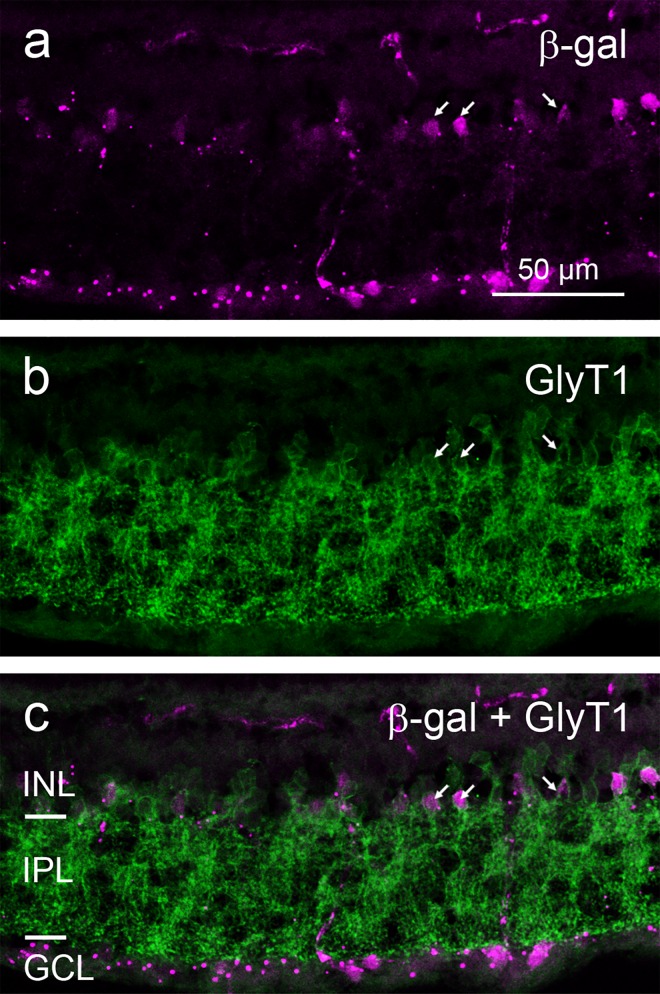
Double immunostaining of a vertical cryostat section of adult FINDT3 mouse retina for β-gal and glycinergic amacrine cells. β-gal label (**a**) colocalizes with the glycine transporter-1 (GlyT1) label (**b**) in some somata in the INL (arrows point to examples), but many glycinergic amacrine cells show no β-gal signal, as evident in the merge (**c**). Images were acquired with an Olympus FluoView 1000 laser scanning microscope.

While in the GCL almost every cell body was stained (see below and discussion), only a fraction of INL cell bodies showed β-gal signal. Based on their location in the inner part of the INL they appeared to be amacrine cells. To confirm this they were co-stained with amacrine cell-specific markers. Most amacrine cells use either GABA or glycine as their transmitter, hence we applied antibodies labeling GABAergic and glycinergic amacrine cells (Figs 2 and 3). Colocalisation with β-gal staining was observed for some GABAergic and glycinergic amacrine cells, although most members of these two populations appeared to be negative for β-gal.

**Fig 4:**
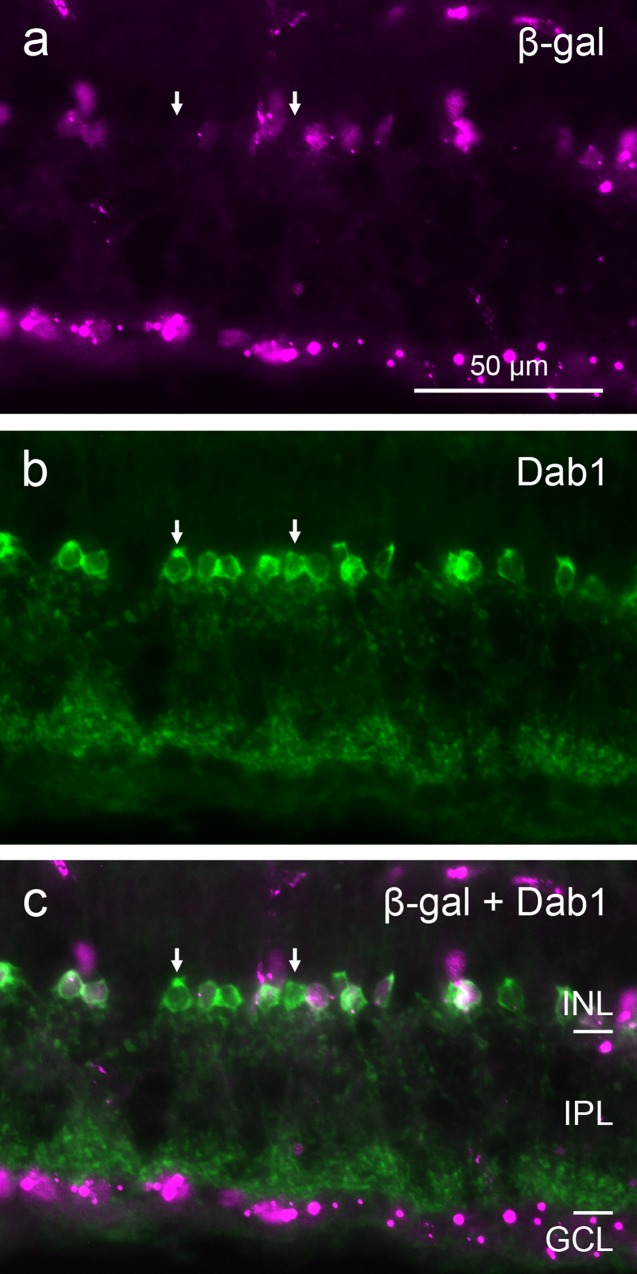
Double immunostaining of a vertical cryostat section of adult FINDT3 mouse retina for β-gal and AII amacrine cells. β-gal label (**a**) colocalizes with the Dab1 label (**b**) in most somata, but some AII cells show no β-gal signal, as evident in the merge (**c**). Two β-gal-negative AII somata are marked by arrows. Images were acquired with a Zeiss Axioplan 2 microscope.

**Fig 5:**
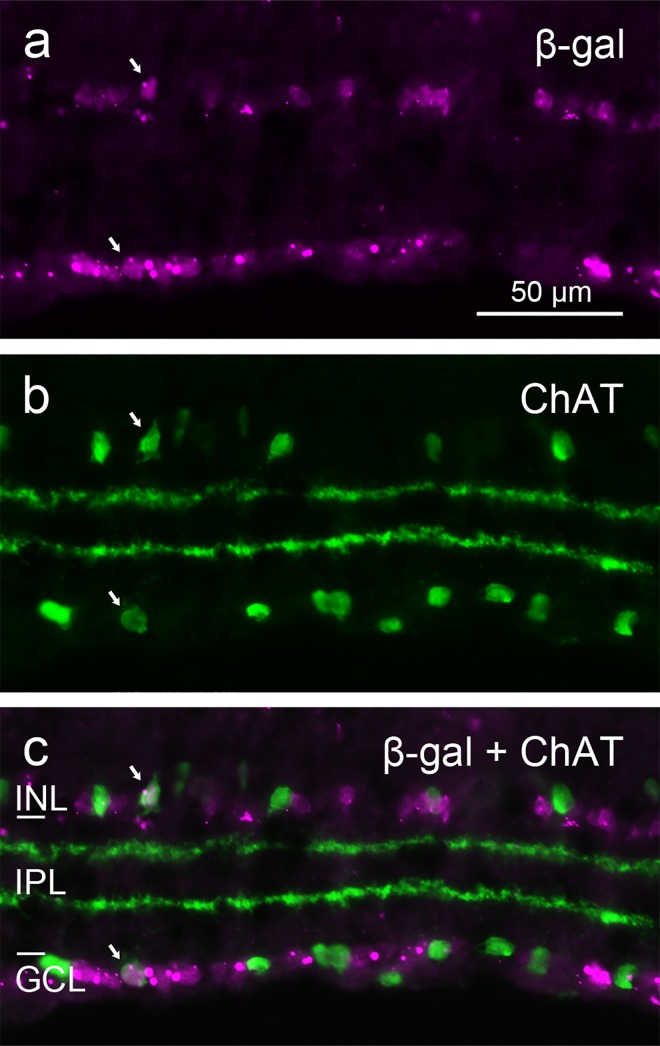
Double immunostaining of a vertical cryostat section of adult FINDT3 mouse retina for β-gal and cholinergic amacrine cells. β-gal label (**a**) colocalizes with the ChAT label (**b**) in a few somata in the INL and GCL (arrows), but the majority of cholinergic amacrine cells show no β-gal signal, as evident in the merge (**c**). Images were acquired with a Zeiss Axioplan 2 microscope.

To assess whether the β-gal-positive cells corresponded to specific cell types among the GABAergic and glycinergic cells, we used type-specific markers that are available for the glycinergic AII amacrines and for the GABA-coexpressing cholinergic ‘starburst’ amacrines. Double-labeling with the AII-specific antiserum against Disabled-1 (Dab1) showed the expected dense population of amacrine cells with the typical AII morphology, of which the majority expressed β-gal (Fig 4). β-gal-negative AII cells constituted a locally varying proportion of less than one third of the AII cells. Double-labeling with an antiserum against choline acetytransferase (ChAT) revealed β-gal signal in a minority of the cholinergic amacrine cell somata in the INL and the GCL (displaced cells), whereas the majority of cholinergic amacrine cells did not show β-gal signal (Fig 5). The proportion of β-gal-positive cholinergic cells varied locally between about 10% and 20%.

**Fig 6:**
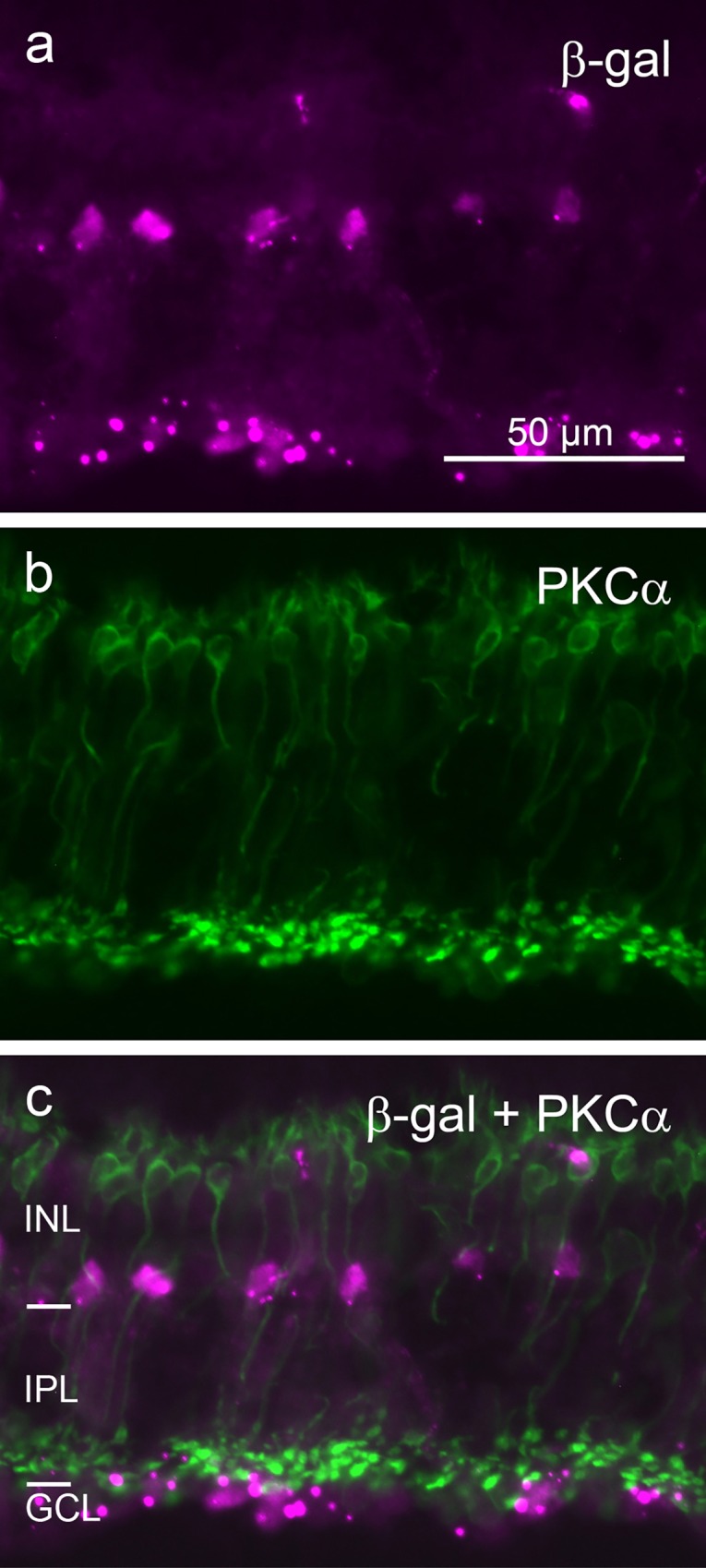
Double immunostaining of a vertical cryostat section of adult FINDT3 mouse retina for β-gal and rod bipolar cells. β-gal label (**a**) does not colocalize with the PKCα label of rod bipolar cells (**b**), as shown in the merge (**c**). The magenta signal among the rod bipolar cell somata in the upper INL represents blood vessel staining. Images were acquired with a Zeiss Axioplan 2 microscope.

**Fig 7:**
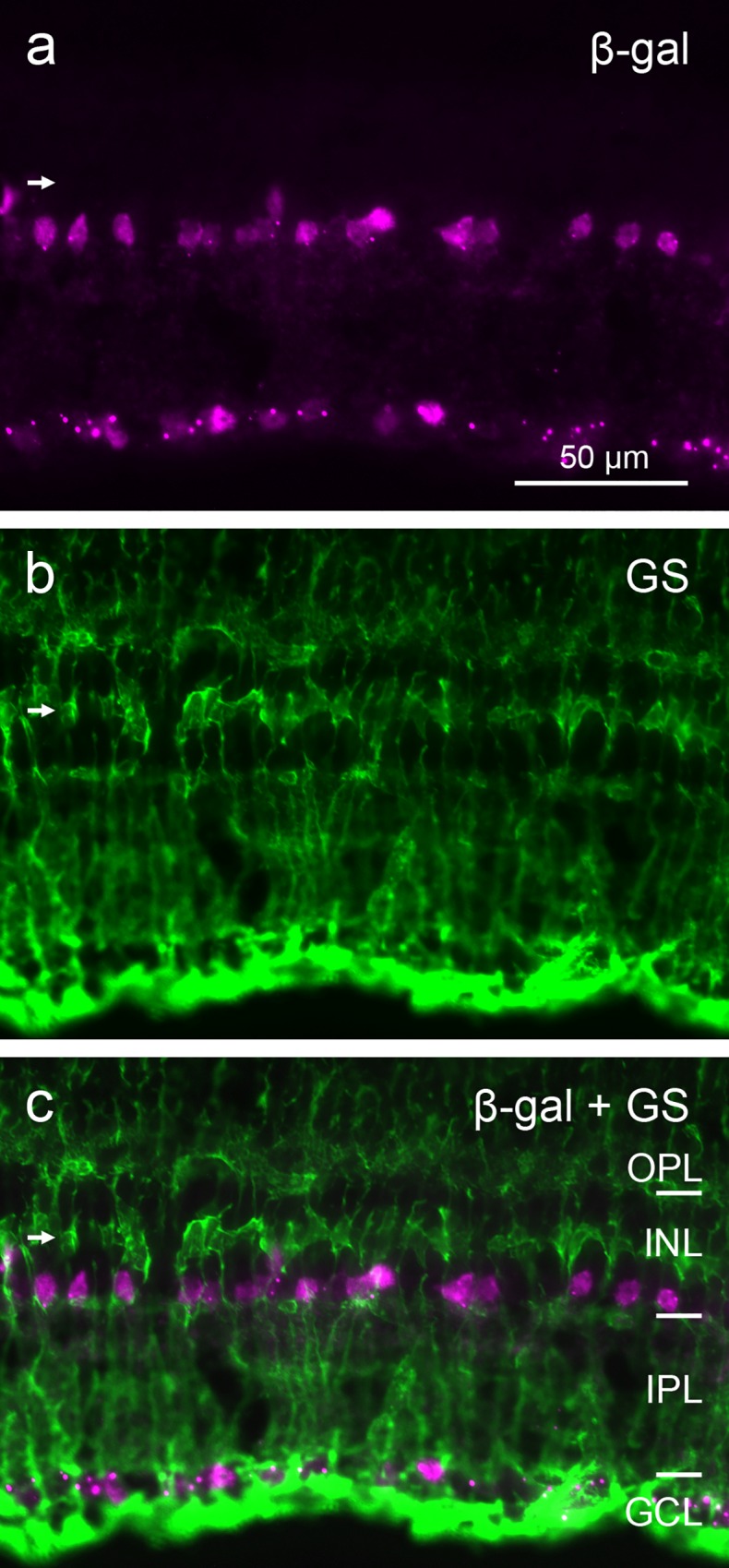
Double immunostaining of a vertical cryostat section of adult FINDT3 mouse retina for β-gal and Müller glia cells. β-gal label (**a**) does not colocalize with the glutamine synthetase label of Müller cells (**b**), as shown in the merge (**c**). The Müller cell processes are stained throughout the retinal layers, the tier of Müller cell somata in the INL is marked by a horizontal arrow. Images were acquired with a Zeiss Axioplan 2 microscope.

To assess whether β-gal label was present in any non-amacrine cells of the INL, we used double-labeling for β-gal with the rod bipolar cell-specific antiserum against PKCα, and with the Müller glia cell-specific antibody against glutamine synthetase. There was no β-gal signal in rod bipolar cells and in Müller cells (Figs 6 and 7). Together these observations confirm that β-gal signal in the INL is limited to subpopulations of amacrine cells.

**Fig 8:**
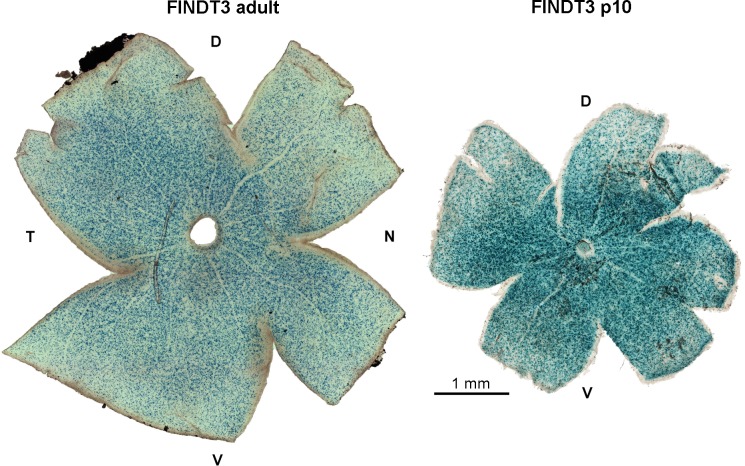
Retinal wholemounts of FINDT3 mice stained with X-gal. Blue dots indicate stained cells in the GCL and INL. **Left**: In the adult retina, the density of stained cells is high in central retina around the optic nerve head and decreases towards the periphery. The density decline is more pronounced in the dorsal part of the retina than in the ventral part. **Right**: At postnatal day p10, the retina shows basically the same density gradient of stained cells as in the adult. D, dorsal; V, ventral; T, temporal; N, nasal. The scale bar applies to both retinae. Images were acquired with a Zeiss Axiophot 2 microscope; the left image is a montage of 20 frames, the right image of 9 frames.

To assess the spatial distribution of β-gal expression in FINDT3 retinae, retinal wholemounts of adult and p10 animals where stained with X-gal (Fig 8). In the adult, the population density of stained GCL cells was highest in central retina around the optic nerve head and decreased towards the retinal periphery, with a slightly steeper density drop towards the dorsal edge of the retina than towards the ventral edge. Basically the same X-gal staining pattern was seen at p10, with an even more pronounced dorsal drop in the density of stained cells. It has to be noted that the staining intensity in the two illustrated retinae cannot be compared, as they result from different incubation and photographic exposure times.

**Fig 9:**
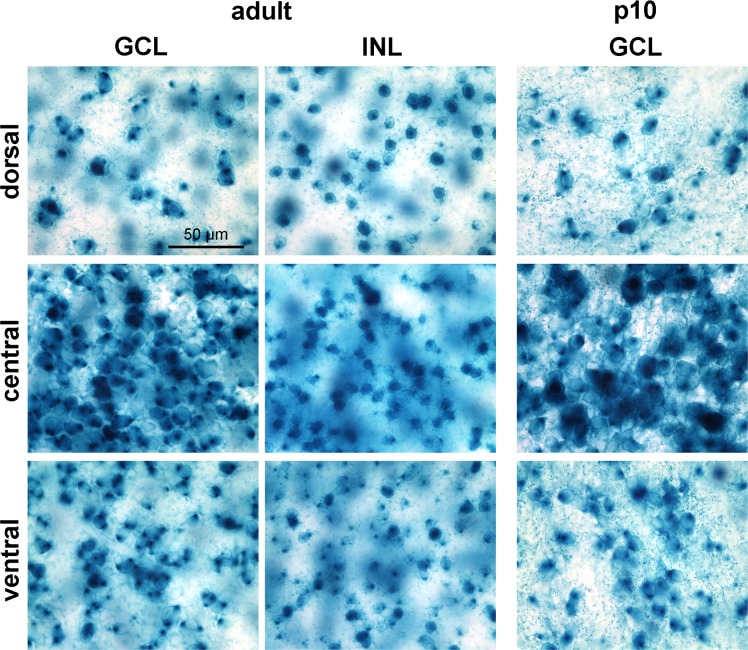
Higher power micrographs from wholemounted FINDT3 retinae stained with X-gal. For the adult mouse (left), the GCL and INL are shown separately as they are on different focal planes. For the p10 mouse (right) only the GCL is shown. Dorsal and ventral fields are from far peripheral retina. The scale bar applies to all images. Images were acquired with a Zeiss Axiophot 2 microscope.

Inspecting the wholemounts at higher magnification revealed more details of the staining pattern in the GCL and INL (Fig 9). The larger somata in the GCL, representing retinal ganglion cells, showed a very strong dot-like labeling in the cytoplasm, often in combination with a weaker and more diffuse labeling of the whole cell body. The smaller somata in the GCL and all somata in the INL, representing amacrine cells, often were more faintly but also more homogeneously labeled. In the p10 retinae, labeled amacrine cells in the INL were very few in numbers (not illustrated).

**Fig 10:**
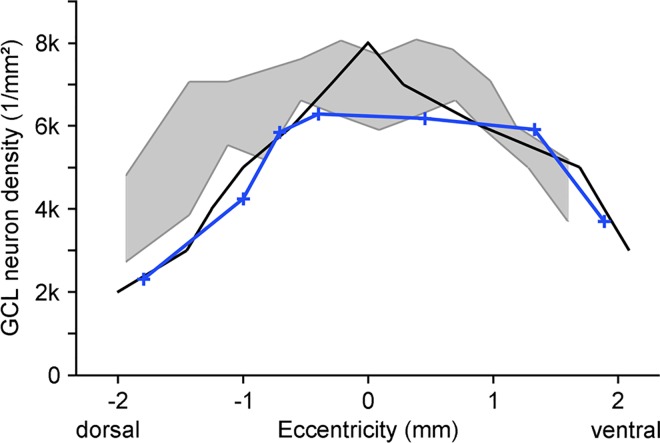
Population densities of ganglion cell layer (GCL) neurons along the dorso-ventral axis of adult mouse retina intersecting the optic nerve head. The blue line gives the densities of X-gal-stained GCL cells in FINDT3 retina. The black line gives the densities of GCL neurons from [32]. The grey area shows the density range of GCL neurons reported for three retinae by [33]. For details see text. Eccentricity ‘0’ refers to the position of the optic nerve head.

To quantify the density distribution for comparison with known cell densities, in a flat-mounted adult retina we counted X-gal-positive cells in the GCL and INL at representative positions along the dorso-ventral axis intersecting the optic nerve head. GCL cells had a high-density plateau of about 6000/mm^2^ in central retina, extending further ventrally than dorsally of the optic nerve head. Peripheral density minima were ca. 3700/mm^2^ ventrally and ca. 2300/mm^2^ dorsally (Fig 10). This density profile is close to that of total GCL neurons, i.e. retinal ganglion cells and displaced amacrine cells, reported in the literature ([32,33]; see discussion). INL cell densities peaked at ca. 5100/mm^2^ in ventral midperiphery, were around 4000/mm^2^ between the optic nerve head and the location of the dorsal drop of GCL cells, and dropped to ca. 3500/mm^2^ in ventral periphery and ca. 2800/mm^2^ in dorsal periphery. Hence the centro-peripheral density gradient of labeled INL cells is somewhat shallower than that of GCL cells and shows a more pronounced density shift to ventral retina.

## Discussion

We have used the FINDT3 reporter mouse to map sites of thyroid hormone signaling in the retina. The reporter plasmid encodes for its own thyroid hormone receptor and is therefore independent of endogenous receptors; the reporter system is most efficient in neurons and much weaker in non-neuronal cells [28]. A major finding of this study is that T3-induced β-gal signal was restricted to cells in the ganglion cell layer (GCL) and to a subset of amacrine cells in the inner nuclear layer (INL). No β-gal signal was detectable in the photoreceptors of the outer nuclear layer (ONL). The absence of reporter activity in cones is surprising, given the pivotal role of thyroid hormone (TH) in controlling cone opsin expression. In the GCL, we found a mild dorso-ventral gradient in β-gal signal, with lower densities of β-gal-positive cells in dorsal than in ventral peripheral retina of both adult and p10 animals. This is the opposite of the TH concentration gradient in p10 mouse retina reported by Roberts et al. [24]. These issues and their potential implications are discussed below.

TH, via its cone-specific receptor TRβ2, has been shown to inhibit S opsin expression and to promote M opsin expression in mouse cones during development [19,21–24,34] and adulthood [25]. Wildtype mice have an unusal cone opsin expression pattern. M opsin expression is high in dorsal retina, where most cones are M cones and only a small fraction are S cones; in ventral retina most cones dominantly express S-opsin and coexpress only low levels of M-opsin [35–37]. It is still open whether the opposing dorso-ventral gradients of S opsin and M opsin expression in the mouse retina originate from a TH gradient [24], or a TRβ2 gradient or both [38], and how they are retained during adulthood. As TRβ2 depends on its ligand TH (particularly T3) for its role in shaping cone opsin identity, all scenarios require the presence of T3 in the cones.

The apparent absence of reporter activity in cones possibly is due to the limited sensitivity of the assay used. Cones downregulate TRβ2, their receptor for thyroid signaling, after postnatal day 5 [20]. Coreceptors for thyroid signaling, necessary for the reporter in the system, may also be downregulated postnatally, resulting in the absence of robust signaling observed at the assayed ages. Available evidence suggests that the primary function of thyroid signaling in cones is opsin choice [19–23], which requires higher levels of signaling after cones are born starting at E15, and low levels of signaling in the adult to maintain opsin expression [25]. Therefore, assaying a time point between E15 and P5 during which TRβ2-mediated signaling is significant should be ideal, but was not feasible within the frame of the present study. We consider it unlikely that β-gal expression in the cones is absent because the reporter system’s Gal4-TRα hybrid receptor misses a specific transcription coactivator. This would imply that TRβ2, which is present and active in the cones, uses coactivators that do not interact with TRα. The existence of such specific coactivators has never been documented in any cell type.

The interpretation that T3 signaling in cones is below the detection threshold of the FINDT3 reporter system also is supported by studies in Dio3^-/-^ mice lacking the T3-inactivating deiodinase Dio3, showing that too high levels of T3 during early postnatal development lead to extensive cone death [26]. Hence it is plausible that T3 in cones is kept at lower levels than in other retinal cells. Tissue fixation parameters also can impede β-gal detection, such that lower β-gal levels remain undetected, even though our fixation times were well below those reported as critical [39]. Furthermore, because thyroid signaling is common, low level signaling would be expected broadly and over-developing the β-gal reaction might result in dim staining in cones but in surrounding rods as well, making cones difficult to distinguish. Given these considerations, the present findings cannot add to the discussion to what extent T3 is available in cones and whether the mouse cone opsin expression gradients correlate with a TH gradient. However, the strong T3 signaling observed in the inner retina suggests that assays based on the measurement of T3 levels in tissue extracts from whole retinae, as performed by Roberts et al [24], might not reflect the situation in cones, as they are likely dominated by the T3 contained in the inner retina.

The size and morphological appearance of the β-gal-positive somata in the GCL indicate that they are neurons, i.e. ganglion cells and displaced amacrine cells, and that non-neuronal cells (glia and blood vessel cells) are not included. This is in line with the fact that the FINDT3 reporter system is most efficient in neurons. Hence for adult mice we can compare the densities of labeled cells with published data on neuron densities in the GCL. There is a qualitative similarity between the dorsal density drop of GCL cells in the X-gal-stained wholemounts (Fig 8) and of mouse retinal ganglion cells ([40]; their figures 3 and 4). For a quantitative comparison, Fig 10 shows the dorso-ventral density profile of β-gal-positive GCL neurons, together with the density profile of total GCL neurons taken from a retinal map in Dräger and Olsen ([32]; their figure 3B) and with the density range of GCL neurons reported for three retinae by Jeon et al. ([33]; their figure 4B). The density profile of the β-gal-positive cells closely follows that of total GCL neurons reported by Dräger and Olsen [32], except for the most central part of the retina. There are ganglion cell density differences between mouse strains [40], and even between different colonies of the same strain [40]. Given this variability, the density profile of β-gal-positive GCL neurons closely approaches the lower margin of total GCL neuron density (Fig 10). We conclude that nearly all neurons in the GCL express β-gal and hence contain T3. The density gradient seen in the wholemounts (Fig 8) reflects the general density gradient of GCL neurons. There appear to be no regional differences in the proportion of GCL neurons that show β-gal signal. In the mouse retina, about 60% of the GCL neurons are displaced amacrine cells and only about 40% are ganglion cells [33], hence a large proportion of the β-gal-positive cells must be displaced amacrine cells (see below). We have not quantified the density of β-gal-positive GCL cells in p10 retinae, because there are no data on overall GCL neuron densities available for comparison. Qualitatively, Figs 8 and 9 show densities comparable to those in the adult retina, suggesting that also at p10 most GCL neurons contain T3.

The β-gal-positive cells in the adult INL are amacrine cells. Their somata are located near the INL/IPL border. This is typical for amacrine cells, whereas the somata of bipolar cells are located in the middle and outer third of the INL, and those of Müller glia cells in the middle of the INL [41–42]. The rod bipolar cells, comprising about 40% of mouse bipolar cells [43], show no β-gal signal (Fig 6). Similarly, the Müller cells are devoid of β-gal signal (Fig 7). β-gal signal in horizontal cells can be excluded, because their somata lie at the outer edge of the INL, where no β-gal signal is seen. The labeling for GABAergic and glycinergic amacrine cells shows that some of these express β-gal. GABA and glycine are the major inhibitory transmitters in the retina and are used by multiple amacrine cell types (review: [44]). The average density of INL cells is 100,000/mm^2^ and the amacrine cells comprise 39% of these, hence their average density is 39,000/mm^2^ [33]. The density range of 5100/mm^2^ to 2800/mm^2^ observed for X-gal-positive amacrine cells is much lower, indicating T3 action in only a minor subset of amacrine cells. In p10 retinae, there were only very few β-gal-positive cells in the INL, showing that many INL amacrine cells acquire their T3 signaling levels later than the GCL neurons.

As the GABAergic and glycinergic amacrine cells each comprise several types, it is possible that β-gal signal, or its lack, is associated with certain amacrine types. We addressed this by labeling two amacrine types for which specific markers are available, the cholinergic ‘starburst’ amacrines and the glycinergic AII amacrines. The cholinergic starburst amacrines are a well-characterized cell type with an important role in creating the direction selectivity of certain ganglion cells (reviews: [45–46]). They consist of two populations, one with somata in the INL, the other with somata in the GCL (displaced starburst cells), and their dendrites form two strata in the IPL. They use acetylcholine and GABA and can be labeled specifically with antibodies against ChAT. Only some of the starburst cell somata in the INL and GCL show β-gal label, whereas the large majority does not (Fig 5). We do not know whether the β-gal-positive cells represent a subtype of starburst cells with continuous β-gal expression (and hence T3 content), or whether β-gal expression in individual starburst cells fluctuates, such that at any one time only some cells appear β-gal-positive. The displaced starburst cells in the GCL constitute 19.5% of all displaced amacrine cells, their density ranges from about 1200/mm^2^ in central retina to about 650/mm^2^ in the periphery ([33]; their figure 6D). As most of these cells do not express β-gal, their density is sufficient to account for the density difference between the total GCL neuron population and the β-gal-positive one seen in Fig 10. This suggests that all other neuron types in the GCL contain T3.

Mouse AII amacrines are labeled specifically with antibodies to disabled-1 (DAB1; [47]). They are involved in the rod pathway, transmitting signals from the rod bipolar cells to the cone bipolar cells (review: [48]). The AII cells form a dense population, and a large proportion shows β-gal labeling and hence T3 signaling (Fig 4). This contrasts with the low proportion of β-gal-positive starburst cells. Nevertheless, both types exemplify heterogeneity of β-gal signal within an amacrine cell type. As stated for the starburst cells above, this poses the question whether the β-gal-positive AII cells represent a subtype with continuous β-gal expression, or whether β-gal expression in individual AII cells fluctuates with time.

The somewhat steeper density decline of β-gal-positive cells, both in the GCL and INL, in dorsal than in ventral peripheral retina indicates that there are more TH-active cells in the ventral than the dorsal half-retina. It is unclear how this fits with the findings of Roberts and colleagues [24], who measured TH levels by a radioimmunoassay in tissue extracts from homogenized dorsal vs. ventral half-retinae of early postnatal mice, and at p10 found about twice as much T3 and T4 (thyroxine) in the dorsal than the ventral half-retina. One explanation is that higher T3 concentrations in the dorsal retina are not resolved by our X-gal staining, which is saturated and not suitable for a quantitative assessment of T3 concentrations.

We here provide the first account of T3 signaling in the inner retina of p10 and mature mouse, as evidenced by β-gal signal in the GCL and INL. It is highly likely that the reporter system in the inner retina specifically indicates receptor-mediated T3 signaling, although ideally in situ hybridization assays for TRβ1 and TRα would be needed to elucidate the mode of signaling. The FINDT3 reporter requires the presence of the ligand T3, delivered by the blood stream, and a coactivator of thyroid signaling. Because coactivators of thyroid signaling (e.g. SRC-1) are shared with other signaling systems it is conceivable that the reporter activity is due to co-activators associated with other signaling networks acting in these cells. However, FINDT3 does not need retinoid X receptors (RXR) for heterodimerization and is unaffected by retinoic acid [28,49]. From two available transgenic mouse lines, FINDT3B with a transgene copy number of approximately 20 was used in the present study [28]. The retinal distribution of the FINDT3B β-gal signal is consistent with published data on the expression profile of TRα in fish [10] and chicken retina [2,50]. The expression pattern of TRα in the adult mammalian retina is largely unknown but may be similar. As evidenced by mRNA in situ hybridization, mouse embryonic stages E14.5 to E18.5 show TRα expression in postmitotic neurons of the inner retina but not in photoreceptors [9], which if pertained is consistent with the diminishing intensity of β-gal signal from inner to outer retina observed in FINDT3 mice.

What role could strong T3 signaling in the cells of the inner retina play? Studies in rats have shown that pharmacologically induced hypothyroidism during the gestation and suckling period results in reduced eye size, delayed retinal development and morphological abnormalities in photoreceptors as well as in the inner retina, including reduced cell densities in the INL and GCL [11–14]. The time window for these wide-ranging effects of hypothyroidism can be narrowed down to the prenatal period. Pax8^-/-^ mice, in which hypothyroidism only manifests itself postnatally, show normally developed retinal layers and normal cell morphologies; their only known retinal abnormality is in cone opsin expression [23]. So apart from cone opsin specification, there is no evidence for the involvement of T3 in the postnatal morphological maturation of the mouse retina and its cell types, and in the maintenance of the adult retina. Even cell types that are generated postnatally, e.g., bipolar cells and Müller glia cells [51], are not affected in the Pax8^-/-^ mouse [23], and do not show β-gal signal in the FINDT3 mouse. As a caveat it has to be noted that the available data come from light microscopy, and a T3 influence on ultrastructural and physiological properties cannot be excluded. Further studies will be needed to elucidate the role of T3 in the inner retina.
